# TGF-β3 Restrains Osteoclastic Resorption Through Autophagy

**DOI:** 10.3390/bioengineering11121206

**Published:** 2024-11-28

**Authors:** Hui Liao, Yiqin Pan, Yiming Liu, Yuxiao Li, Shiyi Huang, Shan Ding, Qi Xiang

**Affiliations:** 1 State Key Laboratory of Bioactive Molecules and Drug Gability Assessment, Jinan University, No. 855 East Xingye Avenue, Guangzhou 510632, China; 18028636361@163.com (H.L.); pyq360734@163.com (Y.P.); 18852812316@163.com (Y.L.); lyx_edison@163.com (Y.L.); huangshiyiha@163.com (S.H.); tdingshan@jnu.edu.cn (S.D.); 2 Institute of Biomedicine, Jinan University, No. 601 Huangpu Avenue West, Guangzhou 510632, China; 3 Guangdong Provincial Key Laboratory of Bioengineering Medicine, Jinan University, No. 601 Huangpu Avenue West, Guangzhou 510632, China; 4 School of Stomatology, Jinan University, No. 601 Huangpu Avenue West, Guangzhou 510632, China; 5 Engineering Research Center of Artificial Organs and Materials, Department of Materials Science and Engineering, Institute of Biomedical Engineering, Jinan University, Guangzhou No. 601 Huangpu Avenue West, Guangzhou 510632, China

**Keywords:** transforming growth factor 3, osteoclast differentiation, bone marrow macrophages, autophagy, bone injury repair

## Abstract

While TGF-β3 promoted defect healing in a primate baboon skull defect model and patients, it remains unclear whether TGF-β3 affects the formation of osteoclasts and bone resorption between osteogenesis and osteolysis. Analysis of the full transcriptome of hPDLSCs (human periodontal ligament stem cells) revealed that the expression of RANKL was significantly up-regulated after TGF-β3 treatment during osteogenesis, which suggests its involvement in clock-controlled autophagy in bone metabolism. TRAP staining and bone resorption lacunae were used to assess the osteoclasts formed from RANKL-induced differentiated BMMs. During osteoclast differentiation, the characteristics of autophagy regulated by TGF-β3 were observed in BMMs through MDC staining, transmission electron microscopy, and LC3 immunofluorescence. The expression of related genes and proteins were detected on the sixth day in mCherry-EGFP-LC3B lentivirus-transfected BMMs using RT-qPCR and WB. Finally, a trans-well co-culture system was used to evaluate the effects of osteogenic differentiated hPDLSCs treated with TGF-β3 on the osteoclastic differentiation of BMMs. The results showed 10 ng/mL of TGF-β3 significantly suppressed osteoclastic differentiation and bone resorption in BMMs (*p* < 0.05 vs. RANKL). In particular, TGF-β3 augments the expression of LC3-II to stimulate autophagy, consequently restraining osteoclastic resorption. These findings provide a molecular basis and are beneficial to illustrate the potential druggability of TGF-β3 in osteoporotic diseases.

## 1. Introduction

TGF-β3, a dimer composed of a polypeptide chain of 110–140 amino acid residues, is a sub-type of the TGFβ family with a relative molecular mass of 25 kD that is mainly secreted by bone, osteoblasts, and articular cartilage. Ababaikeli Guzalinuer et al. [1] have combined TGF-β3 and dental pulp stem cells (DPSCs) into rabbit mandibular defects to promote peri-implant bone integration and repair [1]. Guo et al. have shown that gelatin particles loaded with rabbit bone marrow mesenchymal stem cells (MSCs) and TGF-β3 can promote osteogenic and cartilage differentiation [2]. Ugo Ripamonti et al. have shown that TGF-β3 can promote defect healing in a primate baboon skull defect model and patients with full-thickness mandibular defects [3,4,5]. However, it is not clear whether TGF-β3 affects the formation of osteoclasts and bone resorption in the balance of osteogenesis and osteoclasts. In previous studies, we found that TGF-β3 promoted the osteogenic differentiation of human periodontal ligament stem cells (hPDLSCs), and hPDLSCs combined with TGF-β3 freeze-dried sponge transplantation resulted in an accelerated healing of calvarial bone defects in rats [6]. In addition, an analysis of the in vivo recruitment of stem cells showed that TGF-β3 can also recruit endogenous mesenchymal stem cells, and the in situ injection of a hydrogel loaded with TGF-β3 and bFGF for sequential release into alveolar bone defects significantly promoted bone defect repair [7].

Osteoclasts are multinucleated cells with bone resorption function derived from the proliferation and differentiation of mononuclear macrophages, which play an important role in maintaining the homeostasis of the skeletal system. The differentiation and function of osteoclasts are mainly regulated by two growth factors—RANKL and M-CSF—which are synthesized and secreted by osteoblasts. M-CSF signaling plays an important role in the survival and proliferation of osteoclast precursors, which can interact with the M-CSF receptor to promote the survival of osteoclast precursors, and RANK is continuously expressed. RANKL is synthesized and secreted by osteoblasts, which can recognize each other by RANK expressed by osteoclast precursor cells to promote osteoclast differentiation [8]. As a highly conserved catabolic process in eukaryotic cells, autophagy plays an important role in maintaining cell homeostasis, repairing stress damage, and proliferation and differentiation. More and more studies have shown that various autophagy-related proteins are involved in regulating the survival, differentiation, and function of osteoclasts.

Autophagy plays dual roles in the regulation of osteoclasts [9]. The autophagy inhibitors 3-methyladenine and chloroquine can both effectively down-regulate the expression of osteoclast-related genes and inhibit osteoclastogenesis and its function [10]. Tong et al. have demonstrated that AMPK signaling pathways could promote OPG to inhibit OC differentiation by inducing autophagy [11]. Studies have shown that the autophagy agonist RAP (rapamycin) can inhibit the bone resorption function of osteoclasts [12]. Furthermore, the up-regulation of autophagy levels can reduce the number of apoptotic osteoclasts [13]. Ke et al. have demonstrated, through both in vivo and in vitro experiments, the anti-apoptotic effect of autophagy during RANKL-induced osteoclastogenesis [14]. Kaempferol inhibits autophagy by degrading autophagy substrate proteins and induces apoptosis during osteoclast differentiation [15]. Osteoprotegerin enhances autophagy through the protein kinase/mTOR pathway and inhibits osteoclast differentiation and bone resorption [11]. Under starvation conditions, G protein-coupled receptor kinase interacting protein 1 promotes autophagy in osteoclasts by disrupting the binding of Beclin-1 and Bcl-2 [16].

In addition to osteoclast differentiation and survival, autophagy plays an important role in regulating the bone resorption function of osteoclasts. Autophagy regulates the bone resorption function by affecting the proliferation and differentiation of osteoclasts. Studies have shown that multiple autophagy-related proteins, including ATG5, ATG7, ATG4B, LC3, and Beclin1, are involved in this process and play an important role in various osteoclast functions, such as fold border generation, secretory activity, and bone resorption [17]. It has been found that the autophagy inhibitors 3-methyladenine (3-MA) and CQ (chloroquine) can reduce the expression of osteoclast-related genes. Deselm C. et al. have found that during the formation of OC in rats with ATG5 and ATG7 gene deletions, the morphology of the fold edge on the OC membrane changed, causing the bone resorption activity of OC to decrease [18]. In addition, abnormalities in the autophagy process of OC can cause functional disorders leading to bone-related diseases, such as osteoporosis. Autophagy regulation has been shown to have therapeutic potential in preventing and treating bone-related diseases. Osteoporosis is a common disease in which bone resorption exceeds bone formation. Glucocorticoids induce the production of osteoclasts by promoting autophagy, leading to the occurrence of osteoporosis, while inhibiting the autophagy of osteoclasts can inhibit bone formation [19]. The autophagy inhibitor chloroquine can also prevent the maturation of osteoclasts by inhibiting autophagy and has potential for the treatment of osteoporosis [20].

Autophagy plays an essential role in bone homeostasis. The autophagy process is included not only in osteoclast reabsorption but also in the process of obtaining energy sources during osteoblast differentiation. Altered autophagy may lead to abnormalities in bone cell homeostasis, triggering a range of diseases.

The full transcriptome data of hPDLSCs (human periodontal ligament stem cells) obtained by our research group indicated that the expression of RANKL was significantly up-regulated after adding TGF-β3 during the induction of osteogenesis compared with the osteogenic induction group, which aroused our interest. Does this imply that TGF-β3 not only promotes the osteogenic differentiation of hPDLSCs but also plays an important role in the process of osteoclast differentiation? Therefore, in this study, we explore how TGF-β3 affects the osteoclast differentiation of BMMs through autophagy, simulate the in vivo environment in vitro, and co-culture BMMs and hPDLSCs to explore the role of TGF-β3 in bone homeostasis. Finally, we found that TGF-β3 could inhibit the osteoclast differentiation of BMMs by promoting autophagy.

## 2. Materials and Methods

### 2.1. Animals

SPF female C57BL/6 mice (15 ± 3 g, certificate no. 44007200102049) were supplied by the Guangdong Medical Laboratory Animal Center (Guangdong, China). The animals were housed under controlled conditions, maintaining a constant temperature (25 ± 2 °C), humidity (55% ± 10%), and a 12 h light/dark cycle. The experimental protocol was approved by the Ethics Review Committee for Animal Experimentation of Jinan University (Ethical Review no. 20200826-11), and all experiments were conducted following the National Institutes of Health Guide for the Care and Use of Laboratory Animals (NIH Publications no. 8023, revised 1996).

### 2.2. Cell Culture

BMMs were isolated from the tibia and femur of 6-week-old female C57BL/6 mice. The extraction method referenced the method of Guo X et al. for isolating rabbit marrow mesenchymal stem cells [2]. BMMs were extracted and cultured in high-sugar DMEM (10% fetal bovine serum, 1% penicillin and streptomycin). After conducting the experiment, BMMs were cultured in α-minimum essential medium (α-MEM) supplemented with 10% fetal bovine serum (FBS) and 1% penicillin and streptomycin (P/S, Gibco, Grand Island, NY, USA). The medium was changed every 2 days and the BMMs were cultured until the second generation for use in the experiment. The control group needed M-CSF (416-ML-010/CF, R&D Systems, Minneapolis, MN, USA, USD, 25 ng/mL) added to the culture medium, the RANKL group needed M-CSF (25 ng/mL) and RANKL (462-TEC-010/CF, R&D Systems, USD, 50 ng/mL) simultaneously, and the TGF treatment group needed M-CSF (25 ng/mL), RANKL (50 ng/mL), and TGF-β3 (Jinan University Biopharmaceutical R&D Center, Guangzhou, China). Chloroquine (CQ, AbMole, Abmole Houston, TX, USA, 20 μM in use) and rapamycin (RAP, Sirolimus, Ak Scientific, Inc., Union City, CA, USA, 10 μM in use) were also used in the experiment. After plating, the cells were cultured for 24 h. A total of 4 h of starvation was conducted before treatment, while medium containing 0.4% FBS was used for the post-treatment culture of cells. The extraction and cultivation methods for hPDLSCs were derived from the reference [6].

### 2.3. RNA-Seq and Expression Trend Analysis

Previous studies have shown that TGF-β3 can significantly promote osteogenic differentiation in hPDLSCs, and it is well known that osteogenesis and osteolysis are closely related. Therefore, we focus on the analysis of osteoclasts in order to understand what happened in terms of osteolysis during osteogenic differentiation. The hPDLSCs were cultured in osteogenic differentiation medium (OM) for 3, 7, and 14 days as the OM group. With the same induction time, TGF-β3 was added to OM additionally to form the OM-TGF-β3 group. hPDLSCs before osteogenic induction culture were considered as day 0, representing the control group. After treatment, the concentration of RANKL was measured using a Human RANKL ELISA Kit (BOSTER, Wuhan, China, Lot: Ek0842), and the total RNA was extracted from different groups using TRIzol reagent (Invitrogen, Carlsbad, CA, USA). cDNA libraries were constructed, and samples were paired-end sequenced with an Illumina HiSeq 2000 platform. Whole-transcriptome sequencing data were mapped to the human genome (hg38) using Hisat2. We used HTseq to count the genes and calculate the reads per kilobase transcriptome per million mapped reads (FPKM), in order to evaluate the gene expression level. Time-series gene expression data were analyzed by STEM on the Omicsmart platform. Gene Ontology (GO) enrichment and enrichment network analysis were conducted using Metascape.

### 2.4. LC3 Immunofluorescence

BMMs were fixed and permeabilized. Following permeabilization, the cells were washed 3 times with PBS, and 100 μL BSA solution (5%) was added to block half of the cells at room temperature for 1 h. After blocking, a diluted LC3 primary antibody (1:200) (L8918, Sigma-Aldrich, Saint Louis, MO, USA) was added and incubated overnight at 4 °C. Subsequently, the cells were washed three times with PBS, then incubated with a donkey anti-rabbit Alexa Fluor^®^ 488 secondary antibody (Sigma, Livonia, MI, USA) in the dark for 40 min. Then, cells were washed 3 times with PBS and incubated with antifade mounting medium containing DAPI (P0126, Beyotime, Shanghai, China) for 5 min. The cells were then observed and imaged using a fluorescence microscope.

### 2.5. TRAP Staining

TRAP staining was conducted according to the instructions of the TRAP staining kit (G1492, Solarbio LIFE SCIENCES, Beijing, China). Pictures were captured under an inverted microscope and Image J (V1.54f) was used to count the number of osteoclasts.

### 2.6. Toluidine Blue Staining

Bovine bone chips (40 μm thickness; Broad Biotechnology, China) were sterilized and placed in 96-well plates. BMMs were seeded into the 96-well plates (8 × 10^3^ cells/well). After 15 days of induction, the cells attached to the bone chips were removed by ultrasound in a solution of 0.25 mol/L ammonium hydroxide. After complete dehydration, 1% toluidine blue (Solarbio LIFE SCIENCES, China) was added for staining and incubated for 5 min. The dye solution was washed off with PBS, and the bone chips were then photographed under a microscope (Olympus, Japan). ImageJ was used to calculate and analyze the area of bone resorption pits.

### 2.7. MDC Staining

BMMs were seeded into a 96-well plate (8 × 10^3^ cells/well) and MDC staining was performed on one plate at 2 and 6 days after induction. For MDC staining, MDC solution (G0170, Sigma-Aldrich, USA) was added into the cell plate and incubated at 37 °C for 1 h. The cells were then fixed with 4% paraformaldehyde for 15 min and washed three times with PBS.

### 2.8. Transmission Electron Microscope

BMMs were plated in 6-well plates (5 × 10^5^ cells/well). On the sixth day of induction, the cells were harvested by scraping and centrifuging. Subsequently, the cells were fixed with 2.5% pentanediol overnight at 4 °C. The next day, the cells were rinsed 3 times, fixed with 1% osmic acid, and subjected to gradient dehydration with acetone. The samples were embedded overnight and transferred to an embedding plate for polymerization on the third day. After the reaction, the samples were sectioned and stained with lead citrate and uranyl acetate. Finally, the sections were rinsed and air-dried before being observed and photographed under a transmission electron microscope (HT7800, Hitachi, Tokyo, Japan, 80 kv).

### 2.9. mCherry-EGFP-LC3B Lentiviral Transfection

After preliminary experiments, the MOI value of the BMMs was determined to be 10. The virus stock solution (Zima Gene, Guiyang, China) was diluted tenfold with 10% FBS-H-DMEM and added dropwise into a 24-well plate (2 × 10^4^ cells/well). The plate was then placed in a cell incubator. After culturing for 12 h, the cell status was observed, the medium containing the virus liquid was removed, and the cells were washed with PBS. Fresh 10% FBS-H-DMEM was added to continue the culture. After 48 h of virus transfection, the presence of cell fluorescence was observed under an inverted fluorescence microscope. When fluorescence was observed, drug treatment was performed, and CQ (20 μM), RAP (10 μM), and TGF-β3 (10 ng/mL) treatment groups were set up, as well as a blank control group, and the medium was changed every 2 d. After induction and culturing for 6 days, the cell plate was taken out, the supernatant was discarded, and an anti-fluorescence quencher containing DAPI was added to stain cell nuclei for 2 min after washing with PBS three times. The cell plate was placed under a fluorescence microscope (OLYMPUS IX70, Tokyo, Japan) to observe the intracellular fluorescence expression.

### 2.10. Extraction of Total RNA and Quantitative Real-Time PCR

Total RNA was extracted separately from BMMs induced for 6 days using a Magen RNA extraction kit (R4111, Magen Biotechnology Co., Ltd., Guangzhou, China). Next, cDNA was synthesized utilizing the Reverse Transcription Kit from Accurate Biotechnology (205311, Accurate Biotechnology, Changsha, China). The expression of autophagy-related genes LC3, p62, and Becn1, as well as osteoclast-related genes Ctsk and Nfatc1 were measured using a SYBR^®^ Green Premix Pro Taq HS qPCR Kit (AG11701, Accurate Biotechnology, China). GAPDH and β-Actin were employed as internal reference genes. Primer sequences are provided in the Supplementary Materials (Table S1). The primers sequences were determined using quantitative real-time PCR.

### 2.11. Western Blot

Western blot analysis was performed according to our established protocols [6]. Specifically, equal amounts of total protein (20 μg/lane) were loaded onto 12% SDS-PAGE gel for electrophoresis with 30% Tris–Glycine. Subsequently, proteins were transferred onto a PVDF membrane (IPVH00010, Sigma-Aldrich, USA) using the semi-dry transfer method (TransBlot^®^ Turbo™ Transfer System, Bio-Rad, Hercules, CA, USA, 90 min, 200 mA). First, 5% skim milk was added, then incubated with the appropriate primary antibody at 4 °C overnight, followed by horseradish peroxidase (HRP)-conjugated secondary antibodies. The antibodies used were LC3 (L8918, Sigma-Aldrich, USA), CTSK (DF6614, Affinity, Tampa, FL, USA), P62 (Q13501, Sigma-Aldrich, USA), and GADPH (60004-1-Ig, Proteintech, Wuhan, China).

### 2.12. Culturing BMMs with hPDLSC Osteogenic Induction Culture Supernatant

The hPDLSCs were seeded in 6-well plates (5 × 10^5^ cells/well) and cultured in a conventional cell incubator. When the cell confluence reached 80–90%, osteogenic induction medium was added, and the osteogenic induction groups were cultured for 14 days. The supernatant was collected, filtered through a 0.22 μm microporous membrane, and then stored in aliquots at −20 °C for later use. The second-passage BMMs were inoculated into 96-well plates (8 × 10^3^ cells/well) with a volume of 100 μL per well and cultured in 5% CO_2_ in a 37 °C incubator for 24 h. The collected hPDLSC osteogenic induction culture supernatants were thawed and divided into control and experimental groups. The experimental group was added with osteoclast induction medium containing 10%, 20%, 30%, 40%, or 50% supernatant from osteogenic induction for 14 days and 10 ng/mL TGF-β3. For the control group, 10% FBS-H-DMEM medium containing only M-CSF was used, and the cell plate was cultured in 5% CO_2_ in a 37 °C incubator after administration. The medium was changed every 2 days, and LC3 immunofluorescence was assessed 6 days after induction.

### 2.13. Co-Culture of BMMs and hPDLSCs

The hPDLSCs were seeded in the lower chamber of a trans-well 24-well plate (2 × 10^4^ cells/well), incubated in 5% CO_2_ at 37 °C for 24 h, and then cultured with osteogenic induction medium for 14 days. The second-generation BMMs were taken and seeded in the small chamber of a trans-well 24-well plate (8 × 10^3^ cells/well) with a volume of 100 μL per well and placed in a 5% CO_2_ 37 °C incubator for 24 h. The adherent BMMs in the trans-well 24-well plate chamber were transferred to the trans-well 24-well plate in which hPDLSCs had been osteogenic for 14 days in order to establish an osteoblast–osteoclast co-culture system. After the osteoblast–osteoclast co-culture system was established, the cells were starved for one day in low-serum medium (2% FBS-H-DMEM) before treatment.

### 2.14. Statistical Analysis

The data are presented as the mean ± standard deviation (*n* ≥ 3). Statistical analysis was conducted using GraphPad Prism 6 software (GraphPad Software Inc., La Jolla, CA, USA). For comparisons involving more than two groups, a one-way ANOVA was employed, followed by Tukey’s HSD post hoc test for multiple comparisons. A significance threshold of *p* < 0.05 was used to determine statistical significance.

## 3. Results

### 3.1. Time-Series Transcriptome Profile of hPDLSCs Stimulated by TGF-β3 During Osteogenic Differentiation

**Figure 1:**
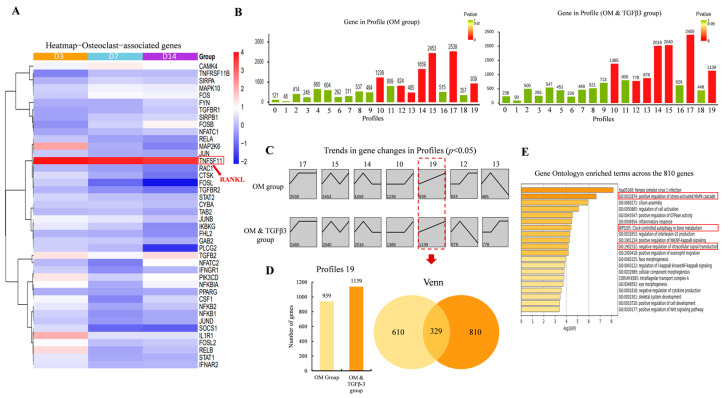
Time-series transcriptome profile of hPDLSCs stimulated by TGF-β3 during osteogenic differentiation. (**A**) Hierarchical clustering heatmap for osteoclast-associated genes. (The alternative name for the gene TNFSF11 is RANKL) (**B**) Differential trends in genes in hPDLSCs during only OM induction; (**C**) differential trends in genes in hPDLSCs during both OM and TGF-β3 induction; (**D**) Venn diagram comparing profile 19 in subfigures (**B**,**C**); and (**E**) heatmap of Gene Ontology enriched terms across the input 810 genes, colored by *p*-values using Metascape (the red box indicates the bone-related signaling pathway).

To explore the possible role of TGF-β3 in the osteogenic differentiation of stem cells, the OM-induced hPDLSCs and the OM-TGF-β3-induced hPDLSCs at 3, 7, and 14 days were analyzed by RNA sequencing. The balance of bone metabolism depends on the balance between bone formation by osteoblasts and bone resorption by osteoclasts. Therefore, in order to investigate the role of TGFβ3 in osteoclast differentiation, we focused on the relevant data of osteoclasts and conducted an in-depth analysis. The osteoclast-associated gene expression heat maps showed that up-regulated expression of the TNFSF11 (TNF Superfamily Member 11, also commonly referred to as osteoclast differentiation factor, RANKL) occurred at days 3, 7, and 14 (Figure 1A). This may be a mechanism of the cell to maintain balance, so it leads to the enhancement of osteogenic differentiation and stimulates the expression of cell osteoclastic differentiation factors. Further analysis of the overall gene expression trends was carried out using STEM (Short Time-series Expression Miner). The results indicated that the change trends of genes could be categorized into 20 profiles, among which profiles 10, 12, 13, 14, 15, 17, and 19 represented significant gene expression trends (*p* < 0.05, Figure 1B,C). Among the significantly clustered profiles, profile 19 contained genes whose abundance showed up-regulated trends across all stages. Between these consistently up-regulated genes, we found 810 genes specific to the TGF-β3 treatment group through a Venn diagram analysis (Figure 1D). Furthermore, the functional enrichment analysis with Metascape found that the 810 genes were significantly enriched in the positive regulation of the stress-activated MAPK cascade, clock-controlled autophagy in bone metabolism, the positive regulation of NIK/NF-kappaB signaling, and so on (Figure 1E), all related to osteoclastogenesis and autophagy.

### 3.2. TGF-β3 Inhibited RANKL-Stimulated Osteoclastogenesis and Bone Resorption Activity of BMMs

**Figure 2:**
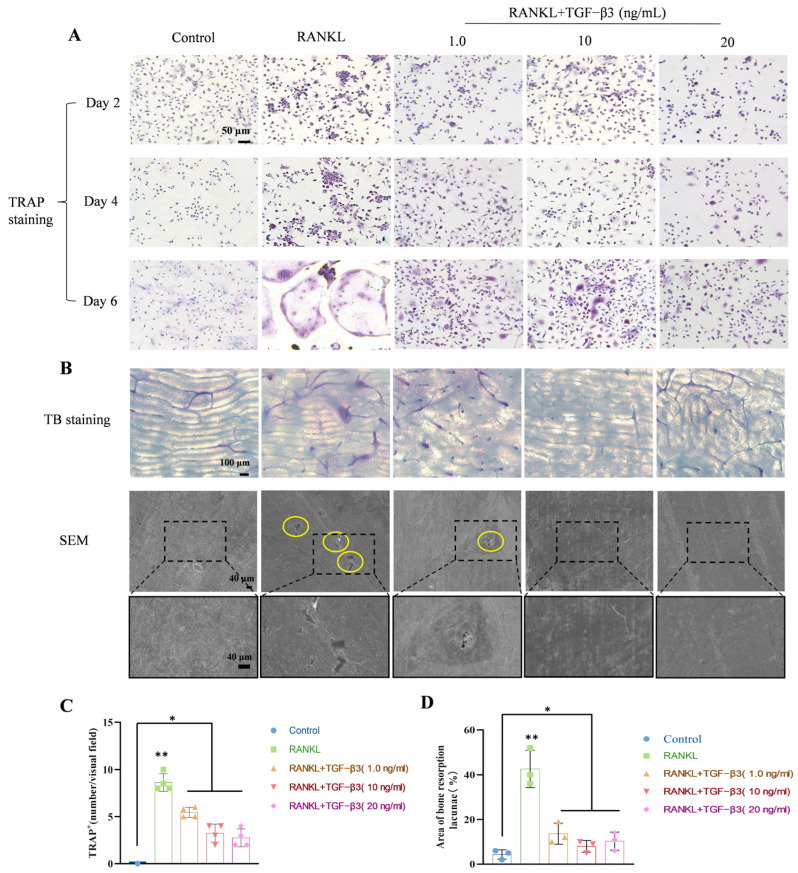
TGF-β3 inhibited RANKL-stimulated osteoclastogenesis and bone resorption activity of BMMs. (**A**) TRAP staining results; (**B**) results of toluidine blue (TB) staining of bone slices and observation of resorption lacunae in bone fragments under scanning electron microscope. Yellow circles represent bone resorption pits; (**C**) number of TRAP+ positive osteoclasts; and (**D**) statistics of bone resorption lacunae (*n* = 3, * *p* < 0.05, ** *p* < 0.01 vs. control).

Figure 2A shows the effect of different concentrations of TGF-β3 on osteoclast differentiation after 6 days. The results of TRAP staining showed that BMMs formed obvious TRAP+ cells with two or more nuclei after being induced for 6 days by RANKL. After treatment with different concentrations of TGF-β3, the number of TRAP+ osteoclasts induced by the sixth day decreased by 62.11% (*p* < 0.05 vs. RANKL), suggesting that TGF-β3 inhibited the differentiation of BMMs into osteoclasts.

After being treated with different concentrations of TGF-β3 for 15 days, the bone fragments were stained with toluidine blue, and the bone resorption pit area was analyzed (Figure 2B). The results showed that no obvious absorption lacuna was formed in the control group. The area of bone resorption lacuna in the RANKL treatment group was larger and the number was larger, while the bone resorption lacuna area in the 10 ng/mL TGF-β3 treatment group was significantly lower than that in the RANKL treatment group (*p* < 0.05). Scanning electron microscope results (Figure 2B) showed little or no obvious bone resorption lacuna in the control group. However, in the bone slices of the RANKL-treated group, obvious bone resorption pits could be seen. After treatment with different concentrations of TGF-β3, the resorption pits on the bone chips were significantly reduced by 81.25% compared with the RANKL-treated group. Figure 2C provides the number of TRAP+ positive osteoclasts, while Figure 2D provides the bone resorption lacunae statistics.

### 3.3. TGF-β3 Promotes Autophagy During Osteoclastic Differentiation of BMMs

**Figure 3:**
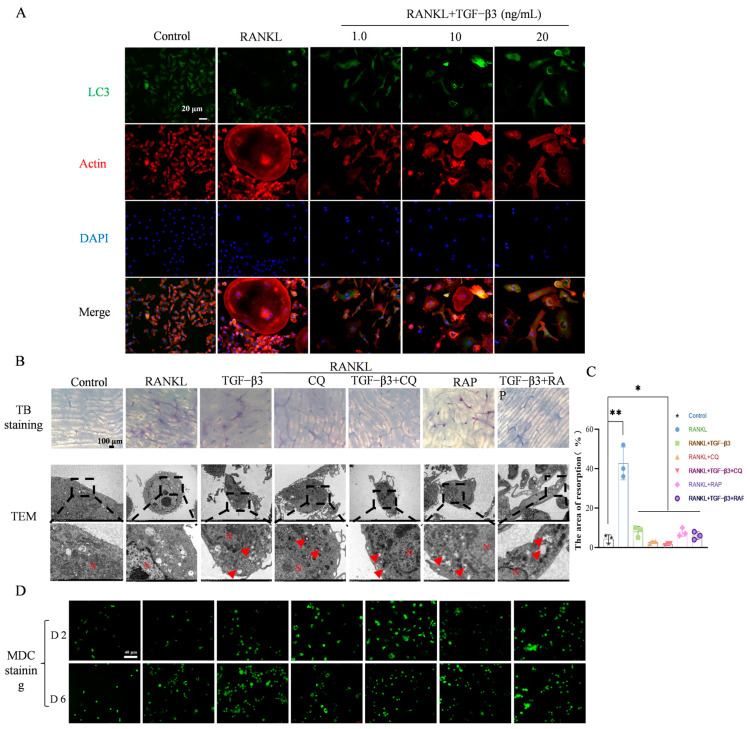
TGF-β3 promotes autophagy during osteoclastic differentiation of BMMs. (**A**) LC3 immunofluorescence shows effect of TGF-β3 on autophagy during osteoclastic differentiation of BMMs; (**B**) results of toluidine blue staining of bone slices and autophagy of BMMs treated with TGF-β3 and CQ for 6 days observed under transmission electron microscope (red arrow indicates autophagolysosome, N indicates nucleus); (**C**) statistics of bone resorption lacunae; and (**D**) MDC staining analysis of autophagy fluorescence changes in BMM cells treated with TGF-β3, CQ, and RAP (*n* = 3, * *p* < 0.05, ** *p* < 0.01 vs. control).

BMMs treated with different concentrations of TGF-β3 were subjected to immunofluorescence staining for autophagy-related protein LC3 in order to detect the distribution and number of LC3 autophagosomes in cells. The immunological results showed (Figure 3A) that compared with the control group, the LC3 fluorescence intensity in the RANKL-treated group was increased to a certain extent, while the fluorescence intensity under the different concentrations of TGF-β3 was higher than that of the RANKL-treated group, with an associated increased number of LC3 autophagosomes. This suggests that the TGF-β3-mediated inhibition of osteolysis is most likely related to autophagy.

After treatment with TGF-β3, CQ, and RAP for 15 days, the bone fragments were stained with toluidine blue and the bone resorption pit area was analyzed (Figure 3B, TB staining). The results indicated that in comparison to the control group (Figure 3C), the size of bone resorption pits increased by 96.07% following RANKL treatment, and there was a significant difference compared with other groups (*p* < 0.05); meanwhile, the TGF-β3 + RAP treatment group presented significantly fewer bone resorption lacunae than the RAP treatment group alone. In addition, autophagosomes were observed by transmission electron microscopy on cells treated with TGF-β3, CQ, and RAP. The results of induction for 6 days (Figure 3B, TEM) showed that compared with the RANKL-treated group, BMMs treated with TGF-β3 had more vesicle-like structures in the cytoplasm (indicated by red arrows (Figure 3B, TEM), further proving that TGF-β3 can induce the accumulation of autophagosomes.

MDC staining was performed on cells treated with TGF-β3, CQ, and RAP for 2 and 6 days, and the results are shown in Figure 3D. Compared with the RANKL-treated group, the MDC fluorescence intensity of TGF-β3-treated BMMs was enhanced. Autophagy inhibitors inhibited autophagy flow, resulting in its obstruction, and the effect in the TGF-β3 + CQ-treated group was higher than that in the CQ-treated group. It is suggested that TGF-β3 can rescue the inhibitory effect of CQ on autophagy. The fluorescence intensity of MDC was enhanced, indicating that TGF-β3 promoted autophagy flow and increased autophagic vesicles.

### 3.4. TGF-β3 Inhibits BMM Osteoclastic Differentiation by Inducing Autophagy

**Figure 4:**
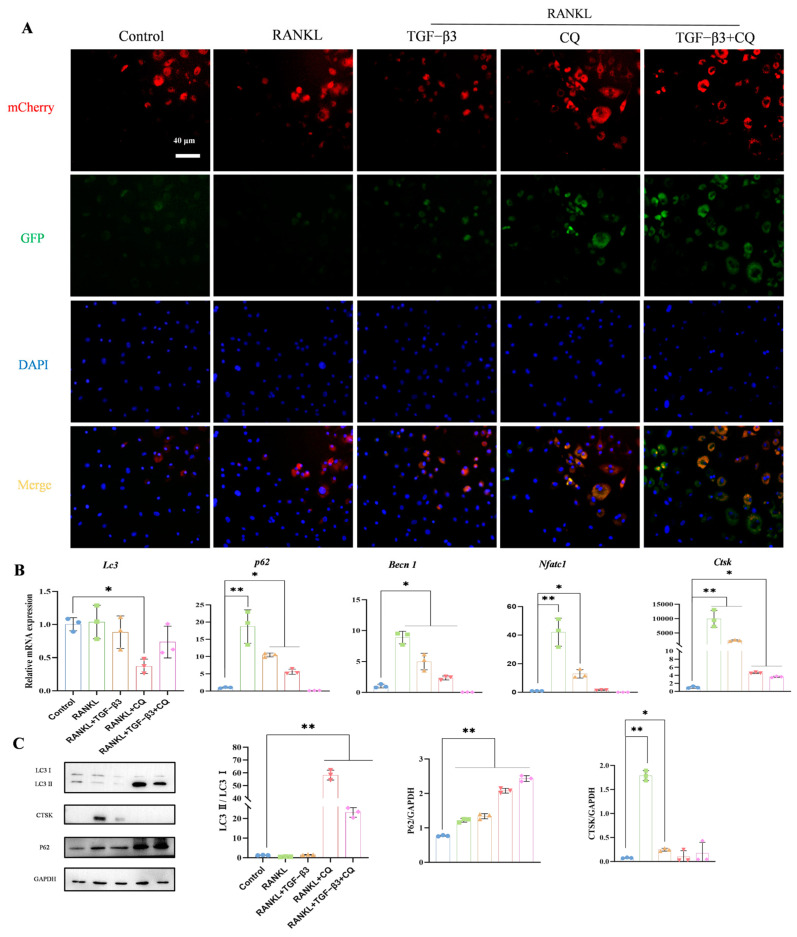
TGF-β3 inhibits osteoclastic differentiation in BMMs by inducing autophagy. (**A**) mCherry-EGFP-LC3B lentivirus was osteoclast-induced for 6 days after successful transfection; (**B**) effects of 6-day treatment with TGF-β3 and CQ on expression of osteoclast- and autophagy-related genes in BMMs; (**C**) expression and semi-quantification of osteoclast- and autophagy-related proteins on day 6 of induced differentiation in BMMs (*n* = 3, * *p* < 0.05, ** *p* < 0.01 vs. control).

To further prove that TGF-β3 promotes autophagic flux and thus increases the number of autophagosomes, BMMs were transfected with mCherry-EGFP-LC3B lentivirus and fluorescence was observed under an inverted microscope. BMMs were successfully transfected with mCherry-EGFP-LC3B lentivirus, and the green fluorescence of GFP and the red fluorescence of mCherry could be seen at 48 h after transfection. At this time, different drug treatments were added, and GFP green fluorescence and mCherry red fluorescence were observed under an inverted microscope after 6 days of induction treatment. After treatment with CQ, the green fluorescence was stronger and after merging with the red fluorescence, it appeared yellow, indicating blockage of the autophagic flow; the green fluorescence was not quenched after treatment with TGF-β3 alone. In contrast, after the addition of TGF-β3 + CQ, the green fluorescence was enhanced and appeared yellow after co-localization with red fluorescence, indicating that TGF-β3 promoted the occurrence of autophagy. However, as CQ is an inhibitor of autophagy in the late stage, it blocked the late stage of autophagy flow, such that GFP fluorescence was not quenched and the fluorescence was enhanced. The results of induction for 6 days are shown in Figure 4A. Compared with the RANKL-treated group, the cells in the TGF-β3-treated group turned red after merging, indicating that the autophagy flow was smooth and the green fluorescence was quenched. The results were similar, indicating that TGF-β3 treatment promoted the occurrence of autophagy but did not affect the development of autophagic flux; as such, the fusion images appear red.

In order to conduct research from the perspective of molecular biology, the expression levels of osteoclast- and autophagy-related genes were detected in BMMs induced for 6 days. RT-qPCR detection showed that after 6 days of induction (Figure 3B) compared with the RANKL treatment group, the expression of the osteoclast-related genes Ctsk and Nfatc1 in the BMMs of the TGF-β3 treatment group was significantly decreased by approximately 76.7% and 72.3%, respectively (*p* < 0.01). Furthermore, compared with the TGF-β3 group, the expression levels of Ctsk and Nfatc1 were significantly decreased by 99.84% and 99.85%, respectively, in the CQ + TGF-β3 treatment group (*p* < 0.01). The expression of the autophagy-related genes LC3 in BMMs in the TGF-β3 treatment group showed no significant difference compared with that in the RANKL treatment group.

WB analysis was conducted to detect the expression of osteoclast- and autophagy-related proteins in BMMs induced for six days. As shown in Figure 4C, compared with the RANKL treatment group, the LC3 II and LC3 I proteins were significantly up-regulated by about 14.2 times in the TGF-β3 treatment group (*p* < 0.01). Furthermore, compared with the CQ treatment group, LC3 II/LC3 I in the TGF-β3 + CQ treatment group were decreased by about 60.9% (*p* < 0.05). Compared with the RANKL treatment group, the expression of CTSK—an osteoclast-related protein—was significantly decreased by 86.6% in the TGF-β3 group (*p* < 0.01). These results suggest that TGF-β3 inhibits osteoclastic differentiation in BMMs by inducing autophagy.

### 3.5. BMMs and hPDLSCs Supernatant Co-Culture and Trans-Well Co-Culture

**Figure 5:**
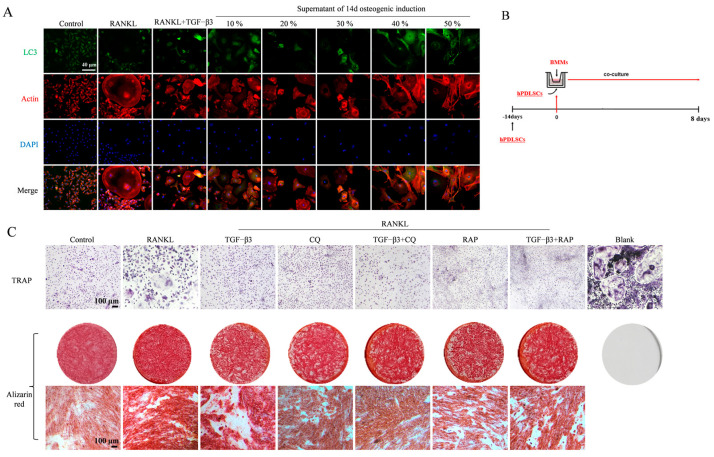
BMM and hPDLSC supernatant co-culture and trans-well co-culture. (**A**) Effect of supernatant of osteogenic induction on BMM autophagy was detected by LC3 immunofluorescence; (**B**) schematic diagram of trans-well co-culture of BMMs and hPDLSCs; and (**C**) BMMs and hPDLSCs co-cultured with trans-well for 8 days. Upper chamber BMMs are stained with TRAP and lower chamber hPDLSCs are stained with Alizarin red.

LC3 immunofluorescence staining was performed on osteoclasts treated with osteogenic induction supernatant at different concentrations (Figure 5A). BMMs treated with RANKL formed obvious osteoclasts, and several cytoskeletons were fused and stretched. LC3 fluorescent staining evidenced obvious spots. After adding different osteogenic induction supernatant treatments, it can be observed that in 40% of the osteogenic supernatant treatment groups, some cells fused to form osteoclast precursor cells and/or presented LC3 fluorescent spots. The number of osteoclasts formed by BMMs in the TGF-β3-treated group was reduced, LC3 aggregation increased, and fluorescence was enhanced. Figure 5B shows a schematic diagram of the trans-well co-culture. After 6 days of co-culture in the trans-well, TRAP staining was performed on BMMs in the upper chamber for the detection of osteoclast differentiation (Figure 5C-TRAP).

The TRAP staining results show that TRAP+ cells appeared in the upper chamber cells of the RANKL-treated group, the cytoplasm of some cells became wine red or purple, the volume was larger, and there were osteoclasts with three or more nuclei, indicating that the cytoplasm of some cells became wine red or purple. Therefore, the co-culture system successfully induced the differentiation of BMMs into mature osteoclasts. Compared with the RANKL-treated group, the number of TRAP+ cells in the TGF-β3-treated group was reduced. Compared with the RAP group, the osteoclasts in the TGF-β3 + RAP group were also reduced.

The Alizarin red staining results (Figure 5C-Alizarin red) for the lower chamber showed that the hPDLSCs in each group had positive reactions after 8 days of co-culture induction. Compared with the normal medium (control), the RANKL treatment group significantly increased the calcium node deposition of hPDLSCs. Compared with the RANKL-treated group, mineral deposition in the TGF-β3 group did not significantly differ. Compared with the RAP treatment group, the hPDLSCs in the TGF-β3 + RAP group had darker Alizarin red staining and more mineral deposition. One can observe that in the trans-well co-culture system, a small amount of TGF-β3-induced autophagy activation could significantly inhibit osteoclastogenesis and promote the late osteogenic differentiation of hPDLSCs compared with the observed autophagy inhibition without an additional differentiation-inducing culture of hPDLSCs. Furthermore, a moderate amount of osteoclast differentiation can promote the late osteogenic differentiation of hPDLSCs.

## 4. Discussion

Osteoclasts are multinucleated giant cells differentiated from mononuclear hematopoietic myeloid cells, which are involved in inflammatory bone destruction. In this experiment, BMMs were used as a cell model, different concentrations of TGF-β3 were added throughout the process of inducing their differentiation into osteoclasts, and the effect of TGF-β3 treatment on the osteoclast differentiation process of BMMs was detected. TRAP is an osteoclast-specific enzyme that exists in the cytoplasm of osteoclasts; thus, TRAP staining is an important indicator for judging osteoclasts. TRAP+ multinucleated cells are cells with three or more nuclei.

TGFβ is a superfamily of polypeptide biological factors which can regulate embryonic growth and development, bone remodeling, cell proliferation and differentiation, and so on. Bitnara Lee et al. have shown that low concentrations of TGFβ can increase the ratio of RANKL/OPG to promote osteoclast differentiation, while high concentrations of TGFβ have the opposite effect on osteoclasts, inhibiting osteoclast differentiation [21]. The results of MTT showed that 10 ng/mL of TGF-β3 had no significant effect on the proliferation of BMMs, while high concentrations of TGF-β3 could inhibit the proliferation of BMMs. Therefore, 1 ng/mL, 10 ng/mL, and 20 ng/mL of TGF-β3 were used in subsequent experiments for the determination of associated functions. The results of the TRAP experiment showed that the 10 ng/mL treatment could significantly inhibit the differentiation of BMMs without affecting the proliferation of BMMs. In this study, a bone slice experiment was used to explore the bone resorption capacity of osteoclasts. The bone slice test results indicated that the bone resorption activity of BMMs treated with 10 ng/mL of TGF-β3 was significantly decreased. Dai Guangming et al. have shown that the down-regulation of TGFβ/Smad4 signaling in osteocytes can increase the expression of OPG, an inhibitor of osteoclast differentiation, thereby inhibiting osteoclast differentiation [22]. In contrast, Asai K et al. have shown that TGFβ can promote osteoclast differentiation in the presence of RANKL and M-CSF [23]. Therefore, the regulatory effect of TGFβ on osteoclasts is still controversial and needs to be further explored.

Autophagy is an evolutionarily conserved intracellular process that digests damaged cells to obtain nutrients and energy [12], which plays an important role in the survival, differentiation, and function of osteoclasts. In the study of autophagy, the intensity of autophagy is often judged according to the fluorescence intensity of MDC, as there exists a positive correlation between the number of MDC fluorescent foci and autophagy activity. Microtubule-associated protein 1A/1B-light chain 3 (LC3) is an important component of autophagy and is incorporated into the inner and outer membranes of autophagosomes during autophagosome biosynthesis. Therefore, LC3 can serve as a specific marker for autophagy, especially the formation of autophagosomes. The MDC staining and LC3 fluorescence results indicated that the immunofluorescence intensity of MDC and LC3 was significantly increased after the treatment of BMMs with TGF-β3, suggesting that TGF-β3 up-regulated autophagy in the process of inhibiting osteoclast differentiation. The TRAP staining and bone chip resorption lacuna experimental results showed that after adding the autophagy inhibitor CQ and autophagy agonist RAP, osteoclast formation and bone resorption activity decreased; meanwhile, after adding RAP + TGF-β3, the bone resorption capacity decreased significantly (*p* < 0.05). Previous studies have shown that autophagy is involved in regulating the formation and bone resorption activity of osteoclasts. For example, LC3 can block the secretion of cathepsin (CTSK) by osteoclasts through the modification of ATG4B, thereby reducing the bone resorption activity of osteoclasts. MDC staining and LC3 immunofluorescence experiments showed that the fluorescence intensity in the TGF-β3 + RAP group was significantly higher than that of RAP alone (*p* < 0.05). Transmission electron microscopy can provide a more intuitive understanding of the formation and morphology of autophagosomes. Under transmission electron microscopy, autophagosomes with a double-membrane vesicle structure of about 500 nm in diameter can be seen, and their double-membrane structure can also be seen. The transmission electron microscopy results demonstrated that the number of autophagosomes in BMMs treated with TGF-β3 was increased compared with that in the RANKL-treated group. p62 can interact with the autophagy-related protein LC3 to be degraded and absorbed by autophagosomes. In this study, it was found that BMMs induced differentiation on the second and sixth days, with the expression of LC3-II higher on the second day than that on the sixth day, while the expression of the p62 protein was lower, indicating that autophagy occurred during osteoclast differentiation. Compared with the RANKL-treated group, LC3II/LC3I in the TGF-β3-treated group were significantly up-regulated (*p* < 0.05). Xu Yiwen et al. have found that in the process of RANKL-induced osteoclast differentiation and maturation, the expression level of the autophagy-related protein LC3 increased, indicating that autophagy plays an important role in RANKL-mediated osteoclast differentiation [24].

The MDC fluorescence, LC3 immunofluorescence, transmission electron microscopy, and Western blot results demonstrated that TGF-β3 induced an alteration in the autophagosomes in BMMs from three aspects, including an increased number, morphology, and autophagy marker proteins. Finally, mCherry-EGFP-LC3B lentivirus was used as a tool to further verify the above conclusions. From the expression of fluorescence intensity after TGF-β3 treatment, it is clear that the green fluorescence in BMMs after TGF-β3 treatment was quenched, indicating that autophagosomes and lysosomes had fused into autophagosomes such that the autophagy flow was smooth. Taken together, this indicates that TGF-β3 promotes autophagy in the process of regulating osteoclast differentiation.

The mechanism of communication regulation between osteoblasts and osteoclasts is a hotspot in bone cell biology research. When exploring the mechanism of bone homeostasis, studies conducted in vitro by culturing osteoblasts or osteoclasts alone cannot reflect the process of bone remodeling, while co-culture systems can better reflect the communication between cells and can simulate cells in the internal environment of bone tissue [16,17,25,26]. Indirect co-culture models can be used to explore paracrine effects between osteoblasts and osteoclasts, such as the release of cytokines, extracellular vesicles, or microRNAs. As osteoblasts secrete a small amount of the TGF-β3 growth factor, in order to explore whether the differentiation of BMMs is inhibited by exogenously added TGF-β3 or the decreased differentiation of BMMs is caused by growth factors secreted by osteoblasts, experiments were carried out in this paper to co-culture BMMs and hPDLSCs. This situation mimics the in vivo bone remodeling microenvironment. The experimental results indicated that the exogenous addition of TGF-β3 inhibited the formation of osteoclasts, and different concentrations of osteogenic induction supernatants had a certain promoting effect on osteoclast differentiation. Sifat Maria et al. have constructed direct and indirect co-culture systems of human mesenchymal stem cells (MSCs) and human peripheral blood mononuclear cells (PBMCs) to mimic the differences in the interactions and communication between osteoblasts and osteoclasts in vivo. It was found that contact with osteoclasts during osteoblastogenesis inhibits RANKL secretion, thereby inhibiting osteoclast formation [27]. Wang Kai et al. have found that the supernatant of RAW264.7 osteoclast differentiation inhibited the proliferation of osteoblasts but promoted osteogenic differentiation and further promoted calcium deposition [28]. The result of this experiment may be that the concentration of TGF-β3 contained in the osteogenic induction supernatant is inconsistent with the concentration of exogenous addition, thus causing different effects on BMMs. Subsequent experiments should focus on detecting the content of TGF-β3 in the supernatant of osteogenic induction or co-cultured hPDLSCs and BMMs in a trans-well in order to further explore the communication between osteoblasts and osteoclasts. Optimizing the osteoblast–osteoclast co-culture model may help to more deeply explore the pathogenesis of bone diseases, which has important clinical significance for exploring potential treatment options for bone diseases [29].

It has been suggested that autophagy plays an important role in the process of bone remodeling [30]. Darcy et al. have reported the induction of autophagy levels using RAP and found that the differentiation of osteoblasts was significantly increased after induction [31]. Future studies should further examine the mechanism of autophagy in fracture repair in detail [32].

The differentiation and maturation of osteoclasts is a complex process regulated by multiple signaling pathways, such as the RANKL–RANK signaling pathway. Recent research has shown that the mTORC1-mediated autophagy signaling pathway may have a bidirectional regulatory effect on OC [33]. Although the experimental results presented in this article showed that TGF inhibits the osteoclastic differentiation of BMMs by activating autophagy, its specific regulatory mechanism on osteoclasts has not yet been clarified, which is also the direction of our future efforts.

The balance of bone metabolism depends on the balance between bone formation by osteoblasts and bone resorption by osteoclasts, and disruption of this balance can lead to a series of diseases related to bone metabolism disorders. Research in recent years has shown that abnormalities in autophagy levels will disrupt the balance of bone metabolism and are related to the occurrence and development of various bone metabolism-related diseases [34], such as osteoporosis (OP), osteoarthritis (OA), Paget’s disease of bone (PDB), and so on. This provides the opportunity to develop novel targets and strategies. It should be noted that autophagy induced by different stages, cells, species, and even different molecules may play different roles; therefore, in the clinical use of autophagy regulators, different judgments need to be made according to individual differences [35]. In the future, we also hope to improve TGF-β3 drugs from the perspective of overall bone metabolism balance, hoping to provide some new treatment ideas for the treatment of diseases related to bone metabolism disorders.

## 5. Conclusions

We observed that treatment with 10 ng/mL of TGF-β3 significantly inhibited the osteoclast differentiation and bone resorption activity of BMMs (*p* < 0.05 vs. RANKL). At the same time, it enhanced the fluorescence intensity of MDC and LC3, which were closely related to autophagy. Under TEM, autophagosomes accumulated markedly. The expression rate of LC3-II/LC3-I in WB also reflected the activation of TGF-β3 in autophagy. In addition, a trans-well co-culture of BMMs and hPDLSCs (pre-induced to osteoblasts for 14 days) revealed that compared with RANKL-induced osteoclasts, TRAP+ cells in the TGF-β3 group were significantly reduced. After the application of TGF-β3 combined with RAP, the autophagy activation effect increased and osteoclast formation decreased. On the other hand, the trace amounts of TGF-β3 in the trans-well co-culture system significantly inhibited osteoclast formation (compared with autophagy inhibition CQ) and simultaneously promoted the late osteogenic differentiation of hPDLSCs. An appropriate amount of osteoclast differentiation helps to promote the late osteogenic differentiation of hPDLSCs. Autophagy is too complex; all kinds of links are intricate and intensive. We just touched on a limited number of biomarkers of autophagy, but it can serve as a guideline. That is, TGF-β3 can inhibit osteoclast differentiation by activating autophagy, which provides new ideas for the treatment of diseases related to bone metabolism disorders.

## Data Availability

The original contributions presented in the study are included in the article; further inquiries can be directed to the corresponding authors.

## Supplementary Materials

The following supporting information can be downloaded at: https://www.mdpi.com/article/10.3390/bioengineering11121206/s1, Figure S1: MTT assay was used to detect the effect of different concentrations of TGF-β3 on the proliferation of BMMs. (n = 3, * *p* < 0.05, vs Control); Figure S2: The autophagy of BMMs treated with TGFβ3 and CQ for 2 days was observed by transmission electron microscope; Figure S3: Lentivirus was induced for 2 days after successful transfection; Figure S4: Effects of TGFβ3 and CQ on expression of 2-day osteoclasts and autophagy related genes in BMMs. (n = 3, * *p* < 0.05, ** *p* < 0.01 vs Control); Figure S5: Expression of osteoclasts and autophagy related proteins on day 2 of BMMs induced differentiation. (n = 3, * *p* < 0.05, ** *p* < 0.01, vs Control); Table S1: qPCR primers sequence.
