# Supplementary material for: TGF-β3 Restrains Osteoclastic Resorption Through Autophagy
Source: Bioengineering (Basel). 2024 Nov 28;11(12):1206. doi: 10.3390/bioengineering11121206 (PMC11673033; doi:10.3390/bioengineering11121206)
Supplement: Supplementary file 1 [file bioengineering-11-01206-s001.zip › Figure S4.pdf]

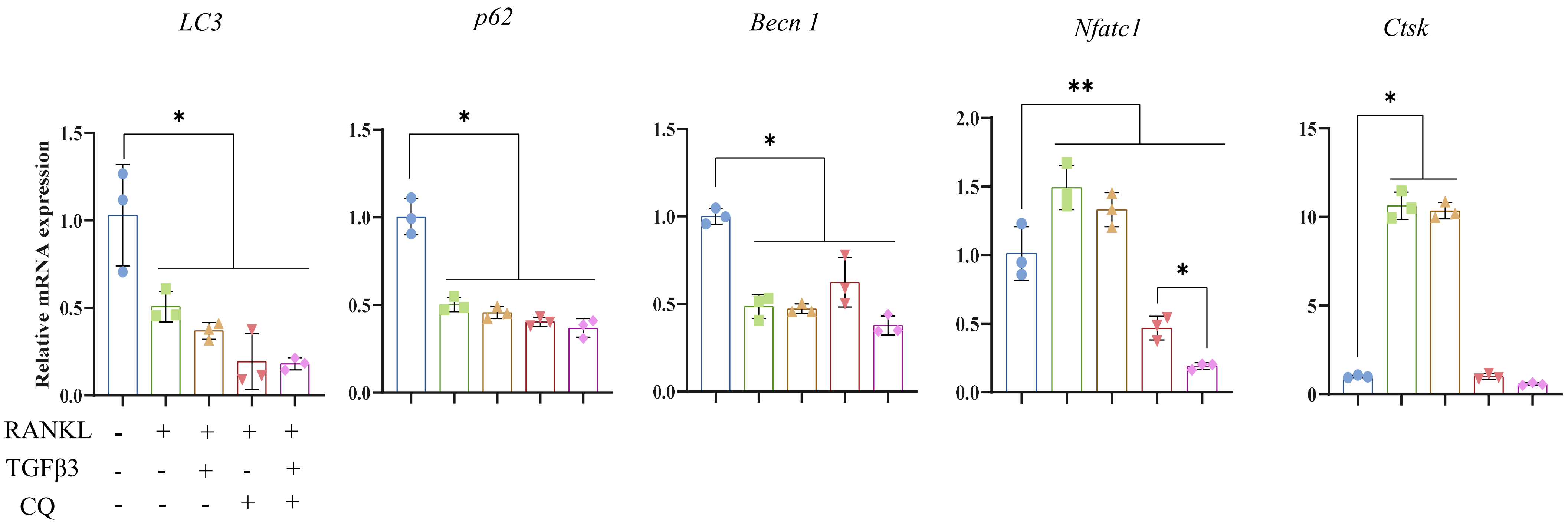

Supplementary Figure 4. Effects of TGFβ3 and CQ on expression of 2-day osteoclasts and autophagy related genes in BMMs. (n=3，*P<0.05, **P<0.01 *vs* Control)
