# Supplementary material for: TGF-β3 Restrains Osteoclastic Resorption Through Autophagy
Source: Bioengineering (Basel). 2024 Nov 28;11(12):1206. doi: 10.3390/bioengineering11121206 (PMC11673033; doi:10.3390/bioengineering11121206)
Supplement: Supplementary file 1 [file bioengineering-11-01206-s001.zip › Table S1.docx]

**Supplementary Materials**

**TGF-β3 restrains osteoclastic resorption through autophagy**

Table S1. qPCR primers sequence

| **Gene** | **Sequence** |
| --- | --- |
| *Nfatc1* | Forward：5’- GAGAATCGAGATCACCTCCTAC-3’ |
|  | Reverse：5’- TTGCAGCTAGGAAGTACGTCTT -3’ |
| *Ctsk* | Forward：CTCGGCGTTTAATTTGGGAGA |
|  | Reverse：TCGAGAGGGAGGTATTCTGAGT |
| *Lc3* | Forward：5′-GCGAGTTGGTCAAGATCATCC-3′ |
|  | Reverse：5′-CCGTCTTCATCCTTCTCCTGTT-3′ |
| *p62* | Forward：5′-CCTTGCCCTACAGCTGAGTC-3′ |
|  | Reverse：5′-TGTTCCACATCAATGTCAACCT-3′ |
| *Becn 1* | Forward：5′-GGGTCACCATCCAGGA-3′ |
|  | Reverse：5′-CACCATCCTGGCGAGTTTCA-3′ |
| *Gapdh* | Forward：5’- GCAAGTTCAACGGCACAG -3’ |
|  | Reverse：5’- CGCCAGTAGACTCCACGAC-3’ |
