# Supplementary figures and images for: TGF-β3 Restrains Osteoclastic Resorption Through Autophagy
Source: Bioengineering (Basel). 2024 Nov 28;11(12):1206. doi: 10.3390/bioengineering11121206 (PMC11673033; doi:10.3390/bioengineering11121206)

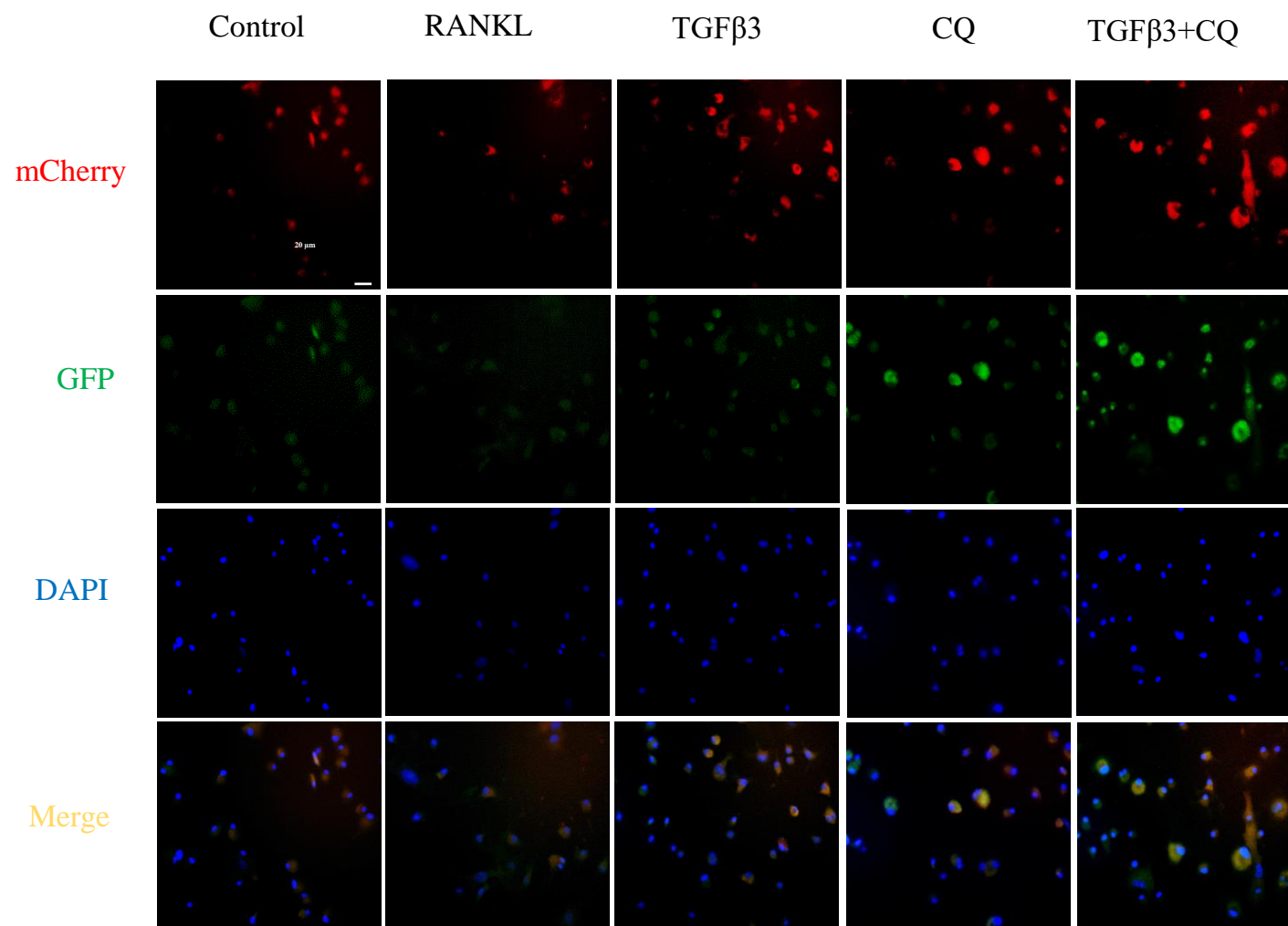

**Supplementary Figure 3.** Lentivirus was induced for 2 days after successful transfection.

Supplement: Supplementary file 1 [file bioengineering-11-01206-s001.zip › Figure S3.pdf]
