# Supplementary material for: TGF-β3 Restrains Osteoclastic Resorption Through Autophagy
Source: Bioengineering (Basel). 2024 Nov 28;11(12):1206. doi: 10.3390/bioengineering11121206 (PMC11673033; doi:10.3390/bioengineering11121206)
Supplement: Supplementary file 1 [file Figure S5.pdf › Figure S5.pdf]

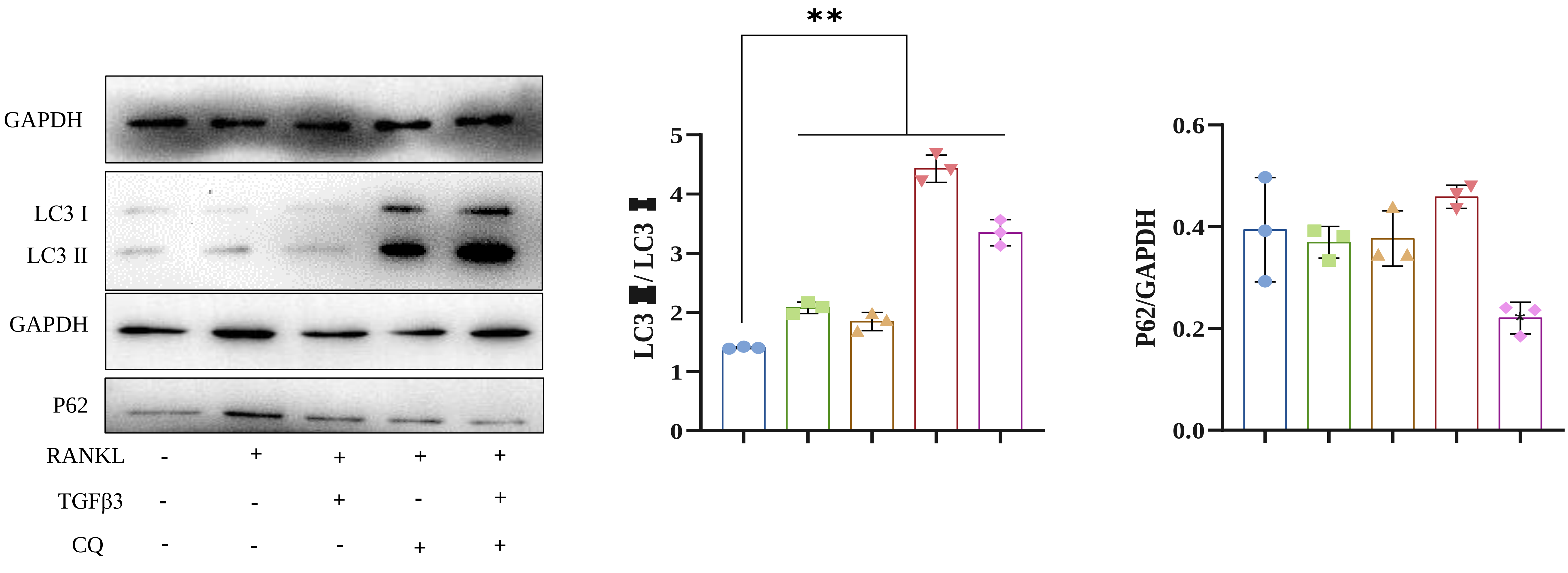

Supplementary Figure 5. Expression of osteoclasts and autophagy related proteins on day 2 of BMMs induced differentiation. (n=3，*P<0.05, **P<0.01, *vs* Control)
